# Chatbot for Health Care and Oncology Applications Using Artificial Intelligence and Machine Learning: Systematic Review

**DOI:** 10.2196/27850

**Published:** 2021-11-29

**Authors:** Lu Xu, Leslie Sanders, Kay Li, James C L Chow

**Affiliations:** 1 Institute of Biomedical Engineering University of Toronto Toronto, ON Canada; 2 Department of Medical Biophysics Western University London, ON Canada; 3 Department of Humanities York University Toronto, ON Canada; 4 Department of English York University Toronto, ON Canada; 5 Department of Medical Physics, Radiation Medicine Program Princess Margaret Cancer Centre University Health Network Toronto, ON Canada; 6 Department of Radiation Oncology University of Toronto Toronto, ON Canada

**Keywords:** chatbot, artificial intelligence, machine learning, health, medicine, communication, diagnosis, cancer therapy, ethics, medical biophysics, mobile phone

## Abstract

**Background:**

Chatbot is a timely topic applied in various fields, including medicine and health care, for human-like knowledge transfer and communication. Machine learning, a subset of artificial intelligence, has been proven particularly applicable in health care, with the ability for complex dialog management and conversational flexibility.

**Objective:**

This review article aims to report on the recent advances and current trends in chatbot technology in medicine. A brief historical overview, along with the developmental progress and design characteristics, is first introduced. The focus will be on cancer therapy, with in-depth discussions and examples of diagnosis, treatment, monitoring, patient support, workflow efficiency, and health promotion. In addition, this paper will explore the limitations and areas of concern, highlighting ethical, moral, security, technical, and regulatory standards and evaluation issues to explain the hesitancy in implementation.

**Methods:**

A search of the literature published in the past 20 years was conducted using the IEEE Xplore, PubMed, Web of Science, Scopus, and OVID databases. The screening of chatbots was guided by the open-access Botlist directory for health care components and further divided according to the following criteria: diagnosis, treatment, monitoring, support, workflow, and health promotion.

**Results:**

Even after addressing these issues and establishing the safety or efficacy of chatbots, human elements in health care will not be replaceable. Therefore, chatbots have the potential to be integrated into clinical practice by working alongside health practitioners to reduce costs, refine workflow efficiencies, and improve patient outcomes. Other applications in pandemic support, global health, and education are yet to be fully explored.

**Conclusions:**

Further research and interdisciplinary collaboration could advance this technology to dramatically improve the quality of care for patients, rebalance the workload for clinicians, and revolutionize the practice of medicine.

## Introduction

### Background

Artificial intelligence (AI) is at the forefront of transforming numerous aspects of our lives by modifying the way we analyze information and improving decision-making through problem solving, reasoning, and learning. Machine learning (ML) is a subset of AI that improves its performance based on the data provided to a generic algorithm from experience rather than defining rules in traditional approaches [1]. Advancements in ML have provided benefits in terms of accuracy, decision-making, quick processing, cost-effectiveness, and handling of complex data [2]. Chatbots, also known as chatter robots, smart bots, conversational agents, digital assistants, or intellectual agents, are prime examples of AI systems that have evolved from ML. The Oxford dictionary defines a chatbot as “a computer program that can hold a conversation with a person, usually over the internet*.*” They can also be physical entities designed to socially interact with humans or other robots. Predetermined responses are then generated by analyzing user input, on text or spoken ground, and accessing relevant knowledge [3]. Problems arise when dealing with more complex situations in dynamic environments and managing social conversational practices according to specific contexts and unique communication strategies [4].

Given these effectual benefits, it is not surprising that chatbots have rapidly evolved over the past 2 decades and integrated themselves into numerous fields, such as entertainment, travel, gaming, robotics, and security. Chatbots have been proven to be particularly applicable in various health care components that usually involve face-to-face interactions. With their ability for complex dialog management and conversational flexibility, integration of chatbot technology into clinical practice may reduce costs, refine workflow efficiencies, and improve patient outcomes [5]. A web-based, self-report survey examining physicians’ perspectives found positive benefits of health care chatbots in managing one’s own health; for improved physical, psychological, and behavioral outcomes; and most notably, for administrative purposes [6]. In light of the opportunities provided by this relatively new technology, potential limitations and areas of concern may arise that could potentially harm users. Concerns regarding accuracy, cybersecurity, lack of empathy, and technological maturity are reported as potential factors associated with the delay in chatbot acceptability or integration into health care [7].

### Objectives

This narrative review paper reports on health care components for chatbots, with a focus on cancer therapy. The rest of this paper is organized as follows: first, we introduce the developmental progress with a general overview of the architecture, design concepts, and types of chatbots; the main *Results* section focuses on the role that chatbots play in areas related to oncology, such as diagnosis, treatment, monitoring, support, workflow efficiency, and health promotion; and the *Discussion* section analyzes potential limitations and concerns for successful implementation while addressing future applications and research topics.

## Methods

This review focuses on articles from peer-reviewed journals and conference proceedings. The following databases were searched from October to December 2020 for relevant and current studies from 2000 to 2020: IEEE Xplore, PubMed, Web of Science, Scopus, and OVID. The literature search used the following key terms: *chatbot*, *chatter robot*, *conversational agent*, *artificial intelligence*, and *machine learning*. For further refinement, these key terms were combined with more specific terms aligned with the focus of the paper. This included *healthcare*, *cancer therapy*, *oncology*, *diagnosis*, *treatment*, *radiation therapy*, and *radiotherapy*. The searches were not limited by language or study design. Letters and technical reports were excluded from the search. The full list of sources and search strategies is available from the authors.

The screening of chatbots was guided by a systematic review process from the Botlist directory during the period of January 2021. This directory was chosen as it was open-access and categorized the chatbots under many different categories (ie, health care, communication, and entertainment) and contained many commonly used messaging services (ie, Facebook Messenger, Discord, Slack, Kik, and Skype). A total of 78 chatbots were identified for health care components and further divided according to the following criteria: diagnosis, treatment, monitoring, support, workflow, and health promotion. It should be noted that using the health filters from a web directory limits the results to the search strategy and marketing label. Thus, the results from equivalent studies may differ when repeated.

## Results

### Chatbot History and Evolution

**Figure 1:**
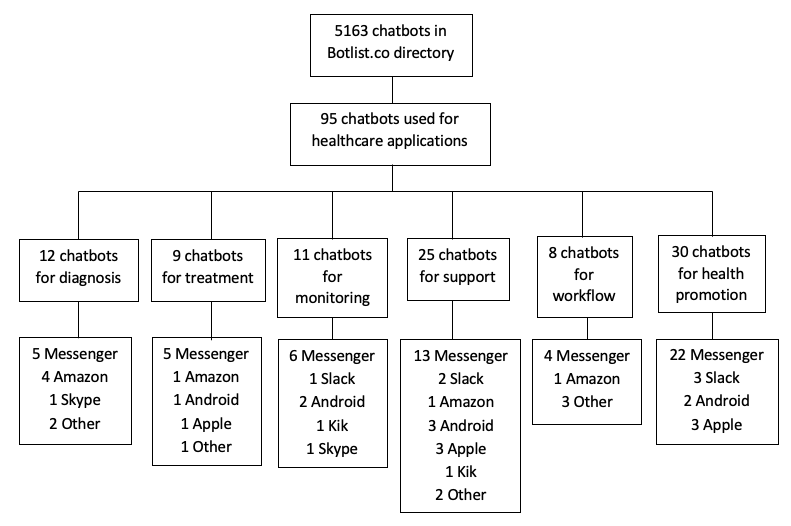
Search and screening for health care chatbots. Chatbots using more than one platform are included.

The idea of a chatbot was first introduced in 1950 when Alan Turing proposed the question, “Can machines think?” [8]. The earliest forms were designed to pass the Turing test and mimic human conversations as much as possible. In 1966, ELIZA (MIT Artificial Intelligence Library) was the first known chatbot developed to act as a psychotherapist, using pattern matching and template-based responses to converse in a question-based format [9]. Improvements were made to build a more human-like and personalized entity by incorporating a personality in PARRY (developed Kenneth Colby) that simulated a paranoid patient [10]. One of the most well-known chatbots is ALICE, developed in 1995 by Richard Wallace, which uses a pattern-matching technique to retrieve example sentences from output templates and avoid inappropriate responses [11]. A renewed interest in AI and advances in ML have led to the growing use and availability of chatbots in various fields [12]. SmarterChild (ActiveBuddy, Inc) [13] became widely accessible through messenger apps, followed by more familiar web-based assistants using voice-activated systems, such as Apple Siri, Amazon Alexa, Google Assistant, and Microsoft Cortana. On the basis of our analysis (Figure 1), the most popular developments of chatbots for health care purposes are diagnostics, patient support (ie, mental health counseling), and health promotion. Some of these applications will be further explored in the following section for cancer applications.

### Chatbot General Architecture

**Figure 2:**
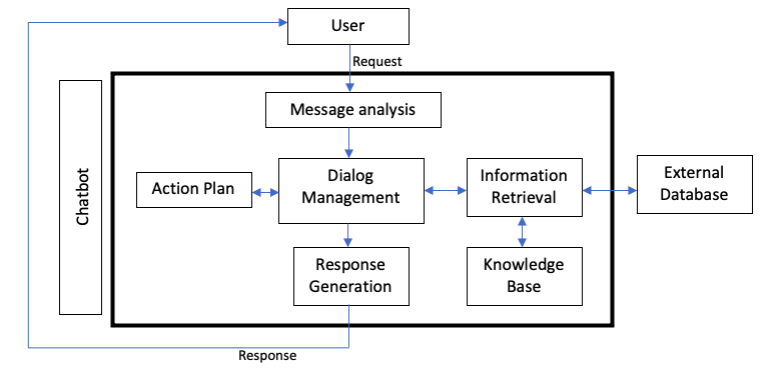
Schematic representation of general chatbot architecture.

Although there are a variety of techniques for the development of chatbots, the general layout is relatively straightforward. As a computer application that uses ML to mimic human conversation, the underlying concept is similar for all types with 4 essential stages (input processing, input understanding, response generation, and response selection) [14]. A simplified general chatbot architecture is illustrated in Figure 2. First, the user makes a request, in text or speech format, which is received and interpreted by the chatbot. From there, the processed information could be remembered, or more details could be requested for clarification. After the request is understood, the requested actions are performed, and the data of interest are retrieved from the database or external sources [15].

### Chatbot Types

With the vast number of algorithms, tools, and platforms available, understanding the different types and end purposes of these chatbots will assist developers in choosing the optimal tools when designing them to fit the specific needs of users. These categories are not exclusive, as chatbots may possess multiple characteristics, making the process more variable. The 5 main types are described below [15]. Textbox 1 describes some examples of the recommended apps for each type of chatbot but are not limited to the ones specified.

Knowledge domain classification is based on accessible knowledge or the data used to train the chatbot. Under this category are the open domain for general topics and the closed domain focusing on more specific information. Service-provided classification is dependent on sentimental proximity to the user and the amount of intimate interaction dependent on the task performed. This can be further divided into interpersonal for providing services to transmit information, intrapersonal for companionship or personal support to humans, and interagent to communicate with other chatbots [14]. The next classification is based on goals with the aim of achievement, subdivided into informative, conversational, and task based. Response generation chatbots, further classified as rule based, retrieval based, and generative, account for the process of analyzing inputs and generating responses [16]. Finally, human-aided classification incorporates human computation, which provides more flexibility and robustness but lacks the speed to accommodate more requests [17].

**Recommended health care components for the different types of chatbots.**

**Knowledge domain**

- Open domain: responding to more general and broader topics that can be easily searched within databases; may be the preferred chatbot type for routine symptom screening, connecting to providers or services, or health promotion apps
- Closed domain: responding to complex or specific questions requiring more in-depth research; may be the preferred chatbot type for treatment planning or recommendation

**Service provided**

- Interpersonal: used mainly to transmit information without much intimate connection with users; may be the preferred chatbot type for imaging diagnostics or hereditary assessment where the main duty is to relay factual information to users
- Intrapersonal: tailored for companionship or support; may be the preferred chatbot type for counseling, emotional support, or health promotion that requires a sense of human touch
- Interagent: used for communicating with other chatbots or computer systems; may be the preferred chatbot type for administration purposes when transferring patient information between locations

**Goal based**

- Informative: designed to provide information from warehouse database or inventory entry; may be the preferred chatbot type for connecting patients with resources or remote patient monitoring
- Conversational: built with the purpose of conversing with users as naturally as possible; may be the preferred chatbot type for counseling, emotional support, or health promotion
- Task based: only performs 1 specific task where actions are predetermined; may be the preferred chatbot type for screening and diagnostics

**Response generation**

- Uses pattern matching when the domain is narrow and sufficient data are available to train the system; may be the preferred chatbot type for screening and diagnostics

**Human aided**

- Incorporates human computation that increases flexibility and robustness but decreases speed; may be the preferred chatbot type for most apps except for support or workflow efficiency, where speed is an essential factor in the delivery of care

### Chatbots in Cancer Therapy

#### Overview

Cancer has become a major health crisis and is the second leading cause of death in the United States [18]. The exponentially increasing number of patients with cancer each year may be because of a combination of carcinogens in the environment and improved quality of care. The latter aspect could explain why cancer is slowly becoming a chronic disease that is manageable over time [19]. Added life expectancy poses new challenges for both patients and the health care team. For example, many patients now require extended at-home support and monitoring, whereas health care workers deal with an increased workload. Although clinicians’ knowledge base in the use of scientific evidence to guide decision-making has expanded, there are still many other facets to the quality of care that has yet to catch up. Key areas of focus are safety, effectiveness, timeliness, efficiency, equitability, and patient-centered care [20].

Chatbots have the potential to address many of the current concerns regarding cancer care mentioned above. This includes the triple aim of health care that encompasses improving the experience of care, improving the health of populations, and reducing per capita costs [21]. Chatbots can improve the quality or experience of care by providing efficient, equitable, and personalized medical services. We can think of them as intermediaries between physicians for facilitating the history taking of sensitive and intimate information before consultations. They could also be thought of as decision aids that deliver regular feedback on disease progression and treatment reactions to help clinicians better understand individual conditions. Preventative measures of cancer have become a priority worldwide, as early detection and treatment alone have not been effective in eliminating this disease [22]. Physical, psychological, and behavioral improvements of underserved or vulnerable populations may even be possible through chatbots, as they are so readily accessible through common messaging platforms. Health promotion use, such as lifestyle coaching, healthy eating, and smoking cessation, has been one of the most common chatbots according to our search. In addition, chatbots could help save a significant amount of health care costs and resources. Newer therapeutic innovations have come with a heavy price tag, and out-of-pocket expenses have placed a significant strain on patients’ financial well-being [23]. With chatbots implemented in cancer care, consultations for minor health concerns may be avoided, which allows clinicians to spend more time with patients who need their attention the most. Costs may also be reduced by delivering medical services more efficiently. For example, the workflow can be streamlined by assisting physicians in administrative tasks, such as scheduling appointments, providing medical information, or locating clinics.

With the rapidly increasing applications of chatbots in health care, this section will explore several areas of development and innovation in cancer care. Various examples of current chatbots provided below will illustrate their ability to tackle the triple aim of health care. The specific use case of chatbots in oncology with examples of actual products and proposed designs are outlined in Table 1.

**Table 1:** Use case for chatbots in oncology, with examples of current specific applications or proposed designs.

Use case and application, chatbot				Function
**Screening and diagnosis**				
	**Imaging diagnostic**			
		Medical Sieve [24]	Examines radiological images to aid clinicians with diagnosis	
	**Symptom screening**			
		Quro [25]	Presynopsis based on symptoms and history to predict user conditions	
		Buoy Health [26]	Assists in identifying the cause of illnesses and provides medical advice	
		Harshitha breast cancer screening [27]	Dialog flow to give an initial analysis of breast cancer symptoms	
		Babylon [28]	Symptom checker	
		Your.md [28]	Symptom checker	
		Ada [28]	Symptom checker	
	**Hereditary assessment**			
		ItRuns [29]	Gathers family history information at the population level to determine the risk of hereditary cancer	
**Treatment**				
	**Patient treatment recommendation**			
		Mathew [30]	Identifies symptoms, predicts the disease using a symptom–disease data set, and recommends a suitable treatment	
		Madhu [31]	Provides a list of available treatments for various diseases and informs the user of the composition and prescribed use of the medications	
	**Connecting patients with providers or resources**			
		Divya [32]	Engages patients regarding their symptoms to provide a personalized diagnosis and connects with appropriate medical service	
		Rarhi [33]	Provides a diagnosis based on symptoms, measures the seriousness, and connects with a physician	
	**Physician treatment planning**			
		Watson for Oncology [34]	Examines data from records and medical notes to generate an evidence-based treatment plan for oncologists	
**Monitoring**				
	**Remote patient monitoring**			
		STREAMD [35]	Provides access to care instructions and educational information	
		Conversa [35]	Provides access to care instructions and educational information	
		Memora Health [35]	Provides access to care instructions and educational information	
		AiCure [36]	Coaches patients to manage their condition and adhere to instructions	
		Infinity [37]	Assesses health outcomes and impact of phone-based monitoring for patients with cancer aged ≥65 years	
		Vik [38,39]	Addresses patients’ daily needs and concerns	
**Support**				
	**Counseling**			
		Vivobot [40]	Cognitive and behavioral intervention for positive psychology skills and promoting well-being	
	**Emotional support**			
		Youper [26]	Daily emotional support and mental health tracking	
		Wysa [26]	Daily emotional support and mental health tracking	
		Replika [26]	Daily emotional support and mental health tracking	
		Unmind [26]	Daily emotional support and mental health tracking	
		Shim [26]	Daily emotional support and mental health tracking	
		Woebot [41]	Daily emotional support and mental health tracking	
**Workflow efficiency**				
	**Administration**			
		Sense.ly [42]	Assists in monitoring appointments, manages patients’ conditions, and suggests therapies	
		Careskore [42]	Tracks vitals and anticipates the need for hospital admissions	
		Mandy [43]	Assists health care staff by automating the patient intake process	
	**Patient encounter**			
		HOLMeS [44]	Supports diagnosis, chooses the proper treatment pathway, and provides prevention check-ups	
**Health promotion**				
	**General lifestyle coaching**			
		SWITCHes [45]	Tracks patients’ progress, provides insight to physicians, and suggests suitable activities	
		CoachAI [46]	Tracks patients’ progress, provides insight to physicians, and suggests suitable activities	
		WeightMentor [47]	Provides self-help motivation for weight loss maintenance and allows for open conversation	
	**Healthy eating**			
		Health Hero [48]	Guides in making informed decisions around food choices to change unhealthy eating habits	
		Tasteful Bot [48]	Guides in making informed decisions around food choices to change unhealthy eating habits	
		Forksy [48]	Guides in making informed decisions around food choices to change unhealthy eating habits	
		SLOWbot [49]	Guides in making informed decisions around food choices to change unhealthy eating habits	
	**Smoking cessation**			
		SMAG [50]	Cognitive behavioral therapy	
		Bella [51]	Coaches to help quit smoking	

#### Diagnostics and Screening

An accurate diagnosis is critical for appropriate care to be administered. In terms of cancer diagnostics, AI-based computer vision is a function often used in chatbots that can recognize subtle patterns from images. This would increase physicians’ confidence when identifying cancer types, as even highly trained individuals may not always agree on the diagnosis [52]. Studies have shown that the interpretation of medical images for the diagnosis of tumors performs equally well or better with AI compared with experts [53-56]. In addition, automated diagnosis may be useful when there are not enough specialists to review the images. This was made possible through deep learning algorithms in combination with the increasing availability of databases for the tasks of detection, segmentation, and classification [57]. For example, Medical Sieve (IBM Corp) is a chatbot that examines radiological images to aid and communicate with cardiologists and radiologists to identify issues quickly and reliably [24]. Similarly, InnerEye (Microsoft Corp) is a computer-assisted image diagnostic chatbot that recognizes cancers and diseases within the eye but does not directly interact with the user like a chatbot [42]. Even with the rapid advancements of AI in cancer imaging, a major issue is the lack of a gold standard [58].

From the patient’s perspective, various chatbots have been designed for symptom screening and self-diagnosis. The ability of patients to be directed to urgent referral pathways through early warning signs has been a promising market. Decreased wait times in accessing health care services have been found to correlate with improved patient outcomes and satisfaction [59-61]. The automated chatbot, Quro (Quro Medical, Inc), provides presynopsis based on symptoms and history to predict user conditions (average precision approximately 0.82) without a form-based data entry system [25]. In addition to diagnosis, Buoy Health (Buoy Health, Inc) assists users in identifying the cause of their illness and provides medical advice [26]. Another chatbot designed by Harshitha et al [27] uses dialog flow to provide an initial analysis of breast cancer symptoms. It has been proven to be 95% accurate in differentiating between normal and cancerous images. Even with promising results, there are still potential areas for improvement. A study of 3 mobile app–based chatbot symptom checkers, Babylon (Babylon Health, Inc), Your.md (Healthily, Inc), and Ada (Ada, Inc), indicated that sensitivity remained low at 33% for the detection of head and neck cancer [28]. The number of studies assessing the development, implementation, and effectiveness are still relatively limited compared with the diversity of chatbots currently available. Further studies are required to establish the efficacy across various conditions and populations. Nonetheless, chatbots for self-diagnosis are an effective way of advising patients as the first point of contact if accuracy and sensitivity requirements can be satisfied.

Early cancer detection can lead to higher survival rates and improved quality of life. Inherited factors are present in 5% to 10% of cancers, including breast, colorectal, prostate, and rare tumor syndromes [62]. Family history collection is a proven way of easily accessing the genetic disposition of developing cancer to inform risk-stratified decision-making, clinical decisions, and cancer prevention [63]. The web-based chatbot ItRuns (ItRunsInMyFamily) gathers family history information at the population level to determine the risk of hereditary cancer [29]. We have yet to find a chatbot that incorporates deep learning to process large and complex data sets at a cellular level. Although not able to directly converse with users, DeepTarget [64] and deepMirGene [65] are capable of performing miRNA and target predictions using expression data with higher accuracy compared with non–deep learning models. With the advent of phenotype–genotype predictions, chatbots for genetic screening would greatly benefit from image recognition. New screening biomarkers are also being discovered at a rapid speed, so continual integration and algorithm training are required. These findings align with studies that demonstrate that chatbots have the potential to improve user experience and accessibility and provide accurate data collection [66].

#### Treatment

Chatbots are now able to provide patients with treatment and medication information after diagnosis without having to directly contact a physician. Such a system was proposed by Mathew et al [30] that identifies the symptoms, predicts the disease using a symptom–disease data set, and recommends a suitable treatment. Although this may seem as an attractive option for patients looking for a fast solution, computers are still prone to errors, and bypassing professional inspection may be an area of concern. Chatbots may also be an effective resource for patients who want to learn why a certain treatment is necessary. Madhu et al [31] proposed an interactive chatbot app that provides a list of available treatments for various diseases, including cancer. This system also informs the user of the composition and prescribed use of medications to help select the best course of action. The diagnosis and course of treatment for cancer are complex, so a more realistic system would be a chatbot used to connect users with appropriate specialists or resources. A text-to-text chatbot by Divya et al [32] engages patients regarding their medical symptoms to provide a personalized diagnosis and connects the user with the appropriate physician if major diseases are detected. Rarhi et al [33] proposed a similar design that provides a diagnosis based on symptoms, measures the seriousness, and connects users with a physician if needed [33]. In general, these systems may greatly help individuals in conducting daily check-ups, increase awareness of their health status, and encourage users to seek medical assistance for early intervention.

Chatbots have also been used by physicians during treatment planning. For example, IBM’s Watson for Oncology examines data from records and medical notes to generate an evidence-based treatment plan for oncologists [34]. Studies have shown that Watson for Oncology still cannot replace experts at this moment, as quite a few cases are not consistent with experts (approximately 73% concordant) [67,68]. Nonetheless, this could be an effective decision-making tool for cancer therapy to standardize treatments. Although not specifically an oncology app, another chatbot example for clinicians’ use is the chatbot Safedrugbot (Safe In Breastfeeding) [69]. This is a chat messaging service for health professionals offering assistance with appropriate drug use information during breastfeeding. Promising progress has also been made in using AI for radiotherapy to reduce the workload of radiation staff or identify at-risk patients by collecting outcomes before and after treatment [70]. An ideal chatbot for health care professionals’ use would be able to accurately detect diseases and provide the proper course of recommendations, which are functions currently limited by time and budgetary constraints. Continual algorithm training and updates would be necessary because of the constant improvements in current standards of care. Further refinements and testing for the accuracy of algorithms are required before clinical implementation [71]. This area holds tremendous potential, as an estimated ≥50% of all patients with cancer have used radiotherapy during the course of their treatment.

#### Patient Monitoring

Chatbots have been implemented in remote patient monitoring for postoperative care and follow-ups. The health care sector is among the most overwhelmed by those needing continued support outside hospital settings, as most patients newly diagnosed with cancer are aged ≥65 years [72]. The integration of this application would improve patients’ quality of life and relieve the burden on health care providers through better disease management, reducing the cost of visits and allowing timely follow-ups. In terms of cancer therapy, remote monitoring can support patients by enabling higher dose chemotherapy drug delivery, reducing secondary hospitalizations, and providing health benefits after surgery [73-75].

StreamMD (StreamMD, Inc), Conversa (Conversa Health, Inc), and Memora Health (Memora Health, Inc) are chatbots that function on existing messaging platforms that provide patients with immediate access to care instructions and educational information [35]. To ensure that patients adhere to instructions, AiCure (AiCure, Inc) uses a smartphone webcam to coach them in managing their condition. Recently, a chatbot architecture was proposed for patient support based on microservices to provide personalized eHealth functionalities and data storage [36]. Several studies have supported the application of chatbots for patient monitoring [76]. The semiautomized messaging chatbot Infinity (Facebook, Inc) was used to assess the health outcomes and health care impacts of phone-based monitoring for patients with cancer aged ≥65 years. After 2 years of implementation, there was a 97% satisfactory rate, and 87% considered monitoring useful, with the most reported benefit being treatment management and moral support [37]. Similar results were discovered in 2 studies using Vik (WeFight, Inc), a text-based chatbot that responds to the daily needs and concerns of patients and their relatives with personal insights. A 1-year prospective study of 4737 patients with breast cancer reported a 94% overall satisfaction rate [38]. A more in-depth analysis of the 132,970 messages showed that users were more likely to answer multiple-choice questions compared with open-ended ones, chatbots improved treatment compliance rate by >20% (*P*=.04), and intimate or sensitive topics were openly discussed. An area of concern is that retention rates drastically decreased to 31% by the end of this study. The other study was a phase 3, blind, noninferiority randomized controlled trial (n=132) to assess the level of patient satisfaction with the answers provided by chatbots versus those by physicians [39]. Using 12 frequently asked questions on breast cancer, participants were split into 2 groups to rate the quality of answers from chatbots or physicians. Among patients with breast cancer in treatment or remission, chatbot answers were shown to be noninferior (*P*<.001), with a success rate of 69% compared with 64% in the physician groups. Concerns regarding the chatbot’s ability to successfully answer more complex questions or detect differences between major and minor symptoms still remain to be addressed.

Further refinements and large-scale implementations are still required to determine the benefits across different populations and sectors in health care [26]. Although overall satisfaction is found to be relatively high, there is still room for improvement by taking into account user feedback tailored to the patient’s changing needs during recovery. In combination with wearable technology and affordable software, chatbots have great potential to affect patient monitoring solutions.

#### Patient Support

The prevalence of cancer is increasing along with the number of survivors of cancer, partly because of improved treatment techniques and early detection [77]. These individuals experience added health problems, such as infections, chronic diseases, psychological issues, and sleep disturbances, which often require specific needs that are not met by many practitioners (ie, medical, psychosocial, informational, and proactive contact) [78]. A number of these individuals require support after hospitalization or treatment periods. Maintaining autonomy and living in a self-sustaining way within their home environment is especially important for older populations [79]. Implementation of chatbots may address some of these concerns, such as reducing the burden on the health care system and supporting independent living.

With psychiatric disorders affecting at least 35% of patients with cancer, comprehensive cancer care now includes psychosocial support to reduce distress and foster a better quality of life [80]. The first chatbot was designed for individuals with psychological issues [9]; however, they continue to be used for emotional support and psychiatric counseling with their ability to express sympathy and empathy [81]. Health-based chatbots delivered through mobile apps, such as Woebot (Woebot Health, Inc), Youper (Youper, Inc), Wysa (Wysa, Ltd), Replika (Luka, Inc), Unmind (Unmind, Inc), and Shim (Shim, Inc), offer daily emotional support and mental health tracking [26]. A study performed on Woebot, developed based on cognitive behavioral therapy, showed that depressive symptoms were significantly reduced, and participants were more receptive than in traditional therapies [41]. This agreed with the Shim results, also using the same type of therapy, which showed that the intervention was highly engaging, improved well-being, and reduced stress [82]. When another chatbot was developed based on the structured association technique counseling method, the user’s motivation was enhanced, and stress was reduced [83]. Similarly, a graph-based chatbot has been proposed to identify the mood of users through sentimental analysis and provide human-like responses to comfort patients [84]. Vivobot (HopeLab, Inc) provides cognitive and behavioral interventions to deliver positive psychology skills and promote well-being. This psychiatric counseling chatbot was effective in engaging users and reducing anxiety in young adults after cancer treatment [40]. The limitation to the abovementioned studies was that most participants were young adults, most likely because of the platform on which the chatbots were available. In addition, longer follow-up periods with larger and more diverse sample sizes are needed for future studies. Chatbots used for psychological support hold great potential, as individuals are more comfortable disclosing personal information when no judgments are formed, even if users could still discriminate their responses from that of humans [82,85].

#### Workflow Efficiency

Electronic health records have improved data availability but also increased the complexity of the clinical workflow, contributing to ineffective treatment plans and uninformed management [86]. A streamlined process using ML techniques would allow clinicians to spend more time with patients by decreasing the time spent on data entry through the ease of documentation, exposing relevant patient information from the chart, automatically authorizing payment, or reducing medical errors [58]. For example, Mandy is a chatbot that assists health care staff by automating the patient intake process [43]. Using a combination of data-driven natural language processing with knowledge-driven diagnostics, this chatbot interviews the patient, understands their chief complaints, and submits reports to physicians for further analysis [43]. Similarly, Sense.ly (Sense.ly, Inc) acts as a web-based nurse to assist in monitoring appointments, managing patients’ conditions, and suggesting therapies. Another chatbot that reduces the burden on clinicians and decreases wait time is Careskore (CareShore, Inc), which tracks vitals and anticipates the need for hospital admissions [42]. Chatbots have also been proposed to autonomize patient encounters through several advanced eHealth services. In addition to collecting data and providing bookings, Health OnLine Medical Suggestions or HOLMES (Wipro, Inc) interacts with patients to support diagnosis, choose the proper treatment pathway, and provide prevention check-ups [44]. Although the use of chatbots in health care and cancer therapy has the potential to enhance clinician efficiency, reimbursement codes for practitioners are still lacking before universal implementation. In addition, studies will need to be conducted to validate the effectiveness of chatbots in streamlining workflow for different health care settings. Nonetheless, chatbots hold great potential to complement telemedicine by streamlining medical administration and autonomizing patient encounters.

#### Health Promotion

Survivors of cancer, particularly those who underwent treatment during childhood, are more susceptible to adverse health risks and medical complications. Consequently, promoting a healthy lifestyle early on is imperative to maintain quality of life, reduce mortality, and decrease the risk of secondary cancers [87]. According to the analysis from the web directory, health promotion chatbots are the most commonly available; however, most of them are only available on a single platform. Thus, interoperability on multiple common platforms is essential for adoption by various types of users across different age groups. In addition, voice and image recognition should also be considered, as most chatbots are still text based.

Healthy diets and weight control are key to successful disease management, as obesity is a significant risk factor for chronic conditions. Chatbots have been incorporated into health coaching systems to address health behavior modifications. For example, CoachAI and Smart Wireless Interactive Health System used chatbot technology to track patients’ progress, provide insight to physicians, and suggest suitable activities [45,46]. Another app is Weight Mentor, which provides self-help motivation for weight loss maintenance and allows for open conversation without being affected by emotions [47]. Health Hero (Health Hero, Inc), Tasteful Bot (Facebook, Inc), Forksy (Facebook, Inc), and SLOWbot (iaso heath, Inc) guide users to make informed decisions on food choices to change unhealthy eating habits [48,49]. The effectiveness of these apps cannot be concluded, as a more rigorous analysis of the development, evaluation, and implementation is required. Nevertheless, chatbots are emerging as a solution for healthy lifestyle promotion through access and human-like communication while maintaining anonymity.

Most would assume that survivors of cancer would be more inclined to practice health protection behaviors with extra guidance from health professionals; however, the results have been surprising. Smoking accounts for at least 30% of all cancer deaths; however, up to 50% of survivors continue to smoke [88]. The benefit of using chatbots for smoking cessation across various age groups has been highlighted in numerous studies showing improved motivation, accessibility, and adherence to treatment, which have led to increased smoking abstinence [89-91]. The cognitive behavioral therapy–based chatbot SMAG, supporting users over the Facebook social network, resulted in a 10% higher cessation rate compared with control groups [50]. Motivational interview–based chatbots have been proposed with promising results, where a significant number of patients showed an increase in their confidence and readiness to quit smoking after 1 week [92]. No studies have been found to assess the effectiveness of chatbots for smoking cessation in terms of ethnic, racial, geographic, or socioeconomic status differences. Creating chatbots with prespecified answers is simple; however, the problem becomes more complex when answers are open. Bella, one of the most advanced text-based chatbots on the market advertised as a coach for adults, gets stuck when responses are not prompted [51]. Therefore, the reaction to unexpected responses is still an area in progress. Given all the uncertainties, chatbots hold potential for those looking to quit smoking, as they prove to be more acceptable for users when dealing with stigmatized health issues compared with general practitioners [7].

## Discussion

### Challenges and Limitations

AI and ML have advanced at an impressive rate and have revealed the potential of chatbots in health care and clinical settings. AI technology outperforms humans in terms of image recognition, risk stratification, improved processing, and 24/7 assistance with data and analysis. However, there is no machine substitute for higher-level interactions, critical thinking, and ambiguity [93]. Chatbots create added complexity that must be identified, addressed, and mitigated before their universal adoption in health care.

Hesitancy from physicians and poor adoption by patients is a major barrier to overcome, which could be explained by many of the factors discussed in this section. A cross-sectional web-based survey of 100 practicing physicians gathered the perceptions of chatbots in health care [6]. Although a wide variety of beneficial aspects were reported (ie, management of health and administration), an equal number of concerns were present. Over 70% of physicians believe that chatbots cannot effectively care for all the patients’ needs, cannot display human emotion, cannot provide detailed treatment plans, and pose a risk if patients self-diagnose or do not fully comprehend their diagnosis. If the limitations of chatbots are better understood and mitigated, the fears of adopting this technology in health care may slowly subside. The *Discussion* section ends by exploring the challenges and questions for health care professionals, patients, and policy makers.

### Moral and Ethical Constraints

The use of chatbots in health care presents a novel set of moral and ethical challenges that must be addressed for the public to fully embrace this technology. Issues to consider are privacy or confidentiality, informed consent, and fairness. Each of these concerns is addressed below. Although efforts have been made to address these concerns, current guidelines and policies are still far behind the rapid technological advances [94].

Health care data are highly sensitive because of the risk of stigmatization and discrimination if the information is wrongfully disclosed. The ability of chatbots to ensure privacy is especially important, as vast amounts of personal and medical information are often collected without users being aware, including voice recognition and geographical tracking. The public’s lack of confidence is not surprising, given the increased frequency and magnitude of high-profile security breaches and inappropriate use of data [95]. Unlike financial data that becomes obsolete after being stolen, medical data are particularly valuable, as they are not perishable. Privacy threats may break the trust that is essential to the therapeutic physician–patient relationship and inhibit open communication of relevant clinical information for proper diagnosis and treatment [96].

Chatbots experience the *Black* *Box* problem, which is similar to many computing systems programmed using ML that are trained on massive data sets to produce multiple layers of connections. Although they are capable of solving complex problems that are unimaginable by humans, these systems remain highly opaque, and the resulting solutions may be unintuitive. This means that the systems’ behavior is hard to explain by merely looking inside, and understanding exactly how they are programmed is nearly impossible. For both users and developers, transparency becomes an issue, as they are not able to fully understand the solution or intervene to predictably change the chatbot’s behavior [97]. With the novelty and complexity of chatbots, obtaining valid informed consent where patients can make their own health-related risk and benefit assessments becomes problematic [98]. Without sufficient transparency, deciding how certain decisions are made or how errors may occur reduces the reliability of the diagnostic process. The *Black Box* problem also poses a concern to patient autonomy by potentially undermining the shared decision-making between physicians and patients [99]. The chatbot’s personalized suggestions are based on algorithms and refined based on the user’s past responses. The removal of options may slowly reduce the patient’s awareness of alternatives and interfere with free choice [100].

Finally, the issue of fairness arises with algorithm bias when data used to train and test chatbots do not accurately reflect the people they represent [101]. As the AI field lacks diversity, bias at the level of the algorithm and modeling choices may be overlooked by developers [102]. In a study using 2 cases, differences in prediction accuracy were shown concerning gender and insurance type for intensive care unit mortality and psychiatric readmissions [103]. On a larger scale, this may exacerbate barriers to health care for minorities or underprivileged individuals, leading to worse health outcomes. Identifying the source of algorithm bias is crucial for addressing health care disparities between various demographic groups and improving data collection.

### Chances for Errors

Although studies have shown that AI technologies make fewer mistakes than humans in terms of diagnosis and decision-making, they still bear inherent risks for medical errors [104]. The interpretation of speech remains prone to errors because of the complexity of background information, accuracy of linguistic unit segmentation, variability in acoustic channels, and linguistic ambiguity with homophones or semantic expressions. Chatbots are unable to efficiently cope with these errors because of the lack of common sense and the inability to properly model real-world knowledge [105]. Another factor that contributes to errors and inaccurate predictions is the large, noisy data sets used to train modern models because large quantities of high-quality, representative data are often unavailable [58]. In addition to the concern of accuracy and validity, addressing clinical utility and effectiveness of improving patients’ quality of life is just as important. With the increased use of diagnostic chatbots, the risk of overconfidence and overtreatment may cause more harm than benefit [99]. There is still clear potential for improved decision-making, as diagnostic deep learning algorithms were found to be equivalent to health care professionals in classifying diseases in terms of accuracy [106]. These issues presented above all raise the question of who is legally liable for medical errors. Avoiding responsibility becomes easier when numerous individuals are involved at multiple stages, from development to clinical applications [107]. Although the law has been lagging and litigation is still a gray area, determining legal liability becomes increasingly pressing as chatbots become more accessible in health care.

### Regulatory Considerations

Regulatory standards have been developed to accommodate for rapid modifications and ensure the safety and effectiveness of AI technology, including chatbots. The US Food and Drug Administration has recognized the distinctiveness of chatbots compared with traditional medical devices by defining the software within the medical device category and has outlined its approach through the Digital Health Innovation Action Plan [108]. With the growing number of AI algorithms approved by the Food and Drug Administration, they opened public consultations for setting performance targets, monitoring performance, and reviewing when performance strays from preset parameters [102]. The American Medical Association has also adopted the Augmented Intelligence in Health Care policy for the appropriate integration of AI into health care by emphasizing the design approach and enhancement of human intelligence [109]. An area of concern is that chatbots are not covered under the Health Insurance Portability and Accountability Act; therefore, users’ data may be unknowingly sold, traded, and marketed by companies [110]. On the other hand, overregulation may diminish the value of chatbots and decrease the freedom for innovators. Consequently, balancing these opposing aspects is essential to promote benefits and reduce harm to the health care system and society.

### Future Directions

Chatbots’ robustness of integrating and learning from large clinical data sets, along with its ability to seamlessly communicate with users, contributes to its widespread integration in various health care components. Given the current status and challenges of cancer care, chatbots will likely be a key player in this field’s continual improvement. More specifically, they hold promise in addressing the triple aim of health care by improving the quality of care, bettering the health of populations, and reducing the burden or cost of our health care system. Beyond cancer care, there is an increasing number of creative ways in which chatbots could be applicable to health care. During the COVID-19 pandemic, chatbots were already deployed to share information, suggest behavior, and offer emotional support. They have the potential to prevent misinformation, detect symptoms, and lessen the mental health burden during global pandemics [111]. At the global health level, chatbots have emerged as a socially responsible technology to provide equal access to quality health care and break down the barriers between the rich and poor [112]. To further advance medicine and knowledge, the use of chatbots in education for learning and assessments is crucial for providing objective feedback, personalized content, and cost-effective evaluations [113]. For example, the development of the Einstein app as a web-based physics teacher enables interactive learning and evaluations but is still far from being perfect [114]. Given chatbots’ diverse applications in numerous aspects of health care, further research and interdisciplinary collaboration to advance this technology could revolutionize the practice of medicine.

On the basis of the discussion above, the following features are general directions of future suggestions for improvements in chatbots within cancer care in no particular order of importance:

1. Patients with cancer may feel vulnerable or fear discrimination from employers or society [115]. Security of sensitive information must be held to the highest standards, especially when personal health information is shared between providers and hospital systems.
2. An increasing number of patients are bringing internet-based information to consultations that are not critically assessed for trustworthiness or credibility. If used correctly, the additional health information could enhance understanding, improve the ability to manage their conditions, and increase confidence during interaction with physicians [116]. Unfortunately, this is often not the case, and most patients are not adequately informed regarding the proper screening of information. Ways to address this challenge include promoting awareness and developing patient management guidelines. Chatbots also have the potential to become a key player in their ability to screen for credible information. They could help vulnerable individuals critically navigate web-based cancer information, especially for the older or more chronic populations that tend to be less technologically adept.
3. Current applications of chatbots as computerized decision support systems for diagnosis and treatment are relatively limited. The targeted audience for most has been for patients’ use, and few are designed to aid physicians at the point of care. Medical Sieve and Watson for Oncology are the only chatbots found in our search that are designed specifically for clinicians. There are far more AI tools in the market to help with clinical decision-making without the ability to interact with users [117]. With the rapid data collection from electronic health records, real-time predictions, and links to clinical recommendations, adding chatbot functionalities to current decision aids will only improve patient-centered care and streamline the workflow for clinicians.
4. More concrete evidence of high quality and accuracy across a broad range of conditions and populations entails more representative training data reflecting racial biases and developing peer-reviewed algorithms to reduce the *Black Box* problem.
5. Integration into the health care system, particularly with telemedicine, for seamless delivery from the beginning to the end does not mean replacing in-person care but rather complementing the health care workflow to ensure patients receive continuity and coordination of care.
6. Reimbursement of chatbot services to physicians who decide to implement this technology into their practice will likely increase adoption rates. Organizations and health providers will likely profit because chatbots allow for a more efficient and reduced cost of delivery.
7. Continual training of chatbots as new knowledge is uncovered, such as symptom patterns or standard of care, is needed.
8. As the Vik study found that users were more likely to respond to multiple-choice questions over open-ended ones [38], chatbot developers should move toward the choice with higher response rates. Studies, surveys, and focus groups should continue to be conducted to determine the best ways to converse with users.
9. Universal adoption of various technical features, such as training with additional languages, image recognition, voice recognition, user feedback to improve services according to needs, access on multiple common platforms, and reacting to unexpected responses, need to be considered.

The ability to accurately measure performance is critical for continuous feedback and improvement of chatbots, especially the high standards and vulnerable individuals served in health care. Given that the introduction of chatbots to cancer care is relatively recent, rigorous evidence-based research is lacking. Standardized indicators of success between users and chatbots need to be implemented by regulatory agencies before adoption. Once the primary purpose is defined, common quality indicators to consider are the success rate of a given action, nonresponse rate, comprehension quality, response accuracy, retention or adoption rates, engagement, and satisfaction level. The ultimate goal is to assess whether chatbots positively affect and address the 3 aims of health care. Regular quality checks are especially critical for chatbots acting as decision aids because they can have a major impact on patients’ health outcomes.

### Review Limitations

The systematic literature review and chatbot database search includes a few limitations. The literature review and chatbot search were all conducted by a single reviewer, which could have potentially introduced bias and limited findings. In addition, our review explored a broad range of health care topics, and some areas could have been elaborated upon and explored more deeply. Furthermore, only a limited number of studies were included for each subtopic of chatbots for oncology apps because of the scarcity of studies addressing this topic. Future studies should consider refining the search strategy to identify other potentially relevant sources that may have been overlooked and assign multiple reviews to limit individual bias.

### Conclusions

As illustrated in this review, these chatbots’ potential in cancer diagnostics and treatment, patient monitoring and support, clinical workflow efficiency, and health promotion have yet to be fully explored. Numerous risks and challenges will continue to arise that require careful navigation with the rapid advancements in chatbots. Consequently, weighing the gains versus threats with a critical eye is imperative. Even after laying down the proper foundations for using chatbots safely and effectively, the human element in the practice of medicine is irreplaceable and will always be present. Health care professionals have the responsibility of understanding both the benefits and risks associated with chatbots and, in turn, educating their patients.

## Abbreviations

AI: artificial intelligence
ML: machine learning
